# Recent advances in methylation modifications of microRNA

**DOI:** 10.1016/j.gendis.2023.101201

**Published:** 2023-12-23

**Authors:** Ning Su, Xiaohang Yu, Ming Duan, Ning Shi

**Affiliations:** State Key Laboratory for Zoonotic Diseases, Key Laboratory for Zoonosis Research of the Ministry of Education, College of Veterinary Medicine, Jilin University, Changchun, Jilin 130062, China

**Keywords:** m^5^C modification, m^6^A modification, m^7^G modification, Methylation modification, miRNA

## Abstract

microRNAs (miRNAs) are short single-stranded non-coding RNAs between 21 and 25 nt in length in eukaryotic organisms, which control post-transcriptional gene expression. Through complementary base pairing, miRNAs generally bind to their target messenger RNAs and repress protein production by destabilizing the messenger RNA and translational silencing. They regulate almost all life activities, such as cell proliferation, differentiation, apoptosis, tumorigenesis, and host–pathogen interactions. Methylation modification is the most common RNA modification in eukaryotes. miRNA methylation exists in different types, mainly N^6^-methyladenosine, 5-methylcytosine, and 7-methylguanine, which can change the expression level and biological mode of action of miRNAs and improve the activity of regulating gene expression in a very fine-tuned way with flexibility. In this review, we will summarize the recent findings concerning methylation modifications of miRNA, focusing on their biogenesis and the potential role of miRNA fate and functions.

## Introduction

microRNAs (miRNAs) are a group of tiny, highly-conserved, single-stranded non-coding RNAs with an average length of 22 nucleotides in eukaryotic organisms. In 1993, Lee et al^1^ and Wightman et al^2^ identified the first miRNA-lin-4 that could regulate gene expression in the model animal nematode. In 2001, when another miRNA, let-7, was discovered, people realized its research value and named this small RNA as miRNA, which means the widespread nature of miRNAs was recognized.^3^ Since then, concerted efforts have been made to discover more miRNAs and illuminate their function began. Currently, a total of 1917 hairpin precursor miRNAs are annotated, leading to the production of 2654 mature miRNAs in the human genome (miRBase, release 22.1); however, the functions of most miRNAs are still unknown.

**Figure 1:**
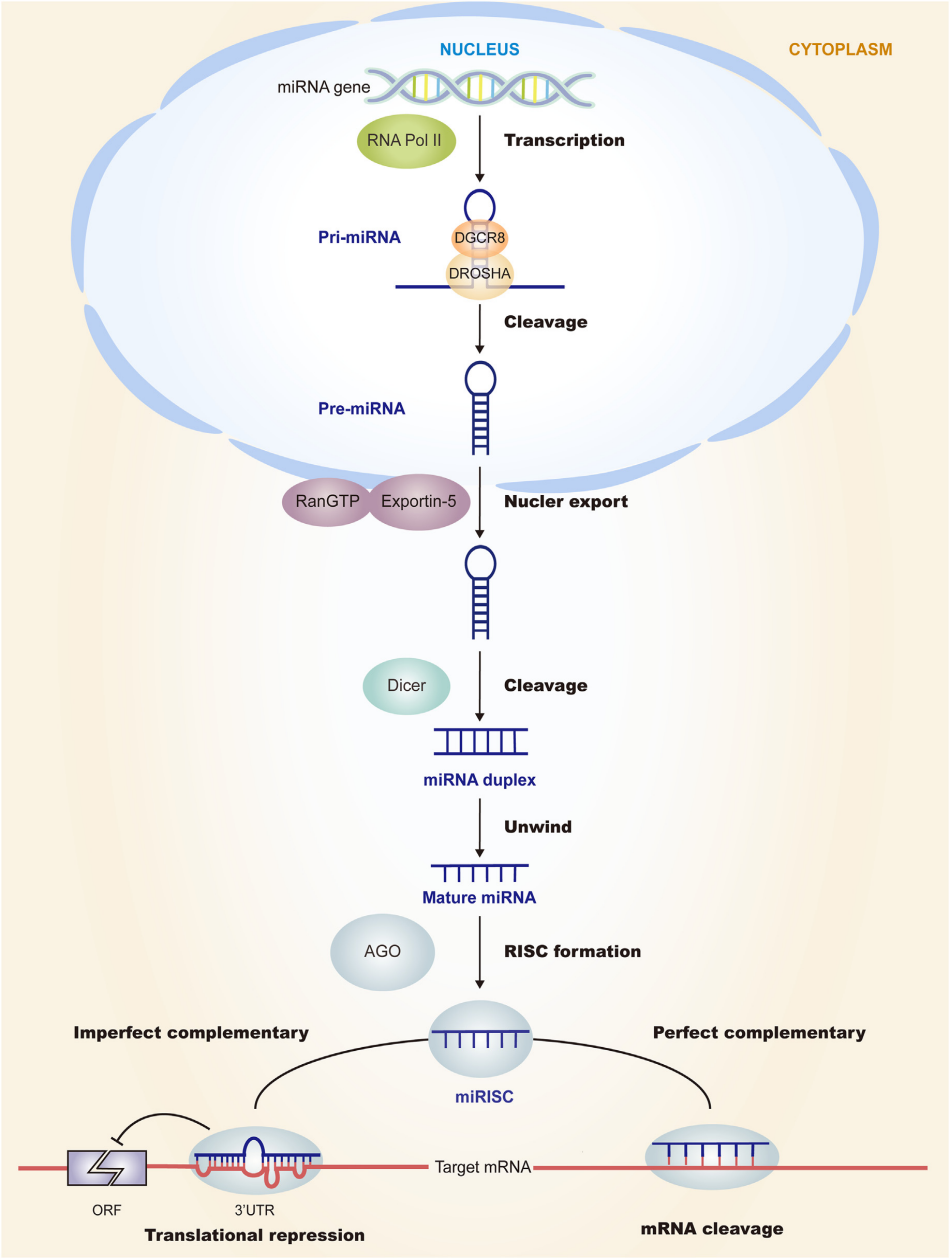
Biosynthesis pathway and function of miRNA. The pri-miRNA is transcribed by RNA polymerase II (Pol II) and cleaved by Drosha/DGCR8 microprocessor complex in the nucleus, which releases pre-miRNA. Then, the pre-miRNA is exported to the cytoplasm by the Exportin5-RanGTP complex. In the cytoplasm, Dicer cleaves the terminal loop of the pre-miRNA to produce the miRNA duplex. The latter is unwound to produce the ∼22-nt-long mature miRNA. Subsequently, the mature miRNA's functional strand is incorporated with Argonaute (AGO) proteins to form the miRISC, playing a role in mRNA decay or translation inhibition. DGCR8, DiGeorge syndrome chromosomal region 8; miRISC, miRNA-induced silencing complex.

The biogenesis of miRNAs has been probed and consists of the following processes (Fig. 1). The majority of miRNAs are transcribed by the RNA polymerase II enzyme, and the production, processing, and assembly of miRNA into the RNA-induced silencing complex (RISC) are all elucidated in earlier research.^4^^,^^5^ miRNAs are first transcribed into primary microRNA (pri-miRNA) precursor molecules in the nucleus, where mature miRNA sequences are ensconced in a stem-loop structure.^6^ Following that, the microprocessor complex, which is made up of the nuclear ribonuclease III-type endonuclease Drosha and its crucial cofactor DiGeorge syndrome chromosomal region 8 (DGCR8), targets and cleaves pri-miRNAs at the stem-loop to create the 65 nt length precursor miRNA (pre-miRNA).^7^, ^8^, ^9^, ^10^ Then, in a way that is dependent on Exportin5 and RanGTP, the pre-miRNA is exported to the cytoplasm.^11^^,^^12^ Once in the cytoplasm, Dicer, another nuclear ribonuclease III endonuclease, converts the double-stranded pre-miRNA into mature miRNAs, one of which is loaded onto the Argonaute (AGO) protein.^13^, ^14^, ^15^ AGO retains the mature miRNA chain and forms an effector complex through RISC formation, called miRNA-induced silencing complex. Upon becoming miRNA-induced silencing complex, the miRNA can either bind perfectly complementarily to the target messenger RNA (mRNA) and cleave it directly, or bind imperfectly complementarily to the 3′-untranslated region (3′-UTR) of the target mRNA and regulate gene expression by mediating translational repression of mRNA, both of which have important implications for post-transcriptional regulation.^16^, ^17^, ^18^, ^19^ According to accumulating data, miRNAs are engaged in nearly all fundamental biological processes, such as cell proliferation, differentiation, metabolism, tumorigenesis, and pathogen-host interactions.^20^^,^^21^

As new, dynamic post-transcriptional controllers of gene expression programs, RNA epigenetic modifications have lately come to light. As early as the 1970s, methylation modification was discovered in eukaryotic RNA.^22^ At this time, cellular RNAs have more than 100 different forms of RNA modifications, many of which are reversible and dynamically controlled.^23^^,^^24^ With the development of RNA sequencing technology, several species of RNA modifications, such as pseudouracil (Ψ) modification,^25^ glycosylation modification,^26^ inosine modification,^27^ and methylation modification,^28^ have been broadly mapped to the transcriptomes of eukaryotic mRNAs and non-coding RNAs.^29^^,^^30^ Accumulating evidence has indicated that post-transcriptional modification of RNA can regulate its charge, base pairing potential, secondary structure, and interactions with proteins, which in turn affect gene expression by modulating RNA stability,^28^^,^^31^ transport,^32^^,^^33^ processing,^34^^,^^35^ and translation.^36^^,^^37^ In addition, few studies suggest that reversible RNA epigenetic modifications have the potential to be used for early intervention and treatment of diseases.

Among hundreds of RNA modifications, methylation modifications are the most numerous and abundant epigenetic modifications in RNA molecules of eukaryotes.^38^ Of the 170 RNA modifications that have been discovered so far, methylation modifications make up more than 70.^39^^,^^40^ Additionally, N-6-methyladenosine (m^6^A) is the considerably more frequent interior post-transcriptional RNA modification in eukaryotic RNAs, such as mRNAs, ribosomal RNAs (rRNAs), transfer RNAs (tRNAs), long noncoding RNAs, and miRNAs.^41^, ^42^, ^43^, ^44^ It is also the earliest methylation modification discovered in eukaryotic RNA. Since then, scientists have also identified other forms of RNA methylation, including N-1-methyladenosine, 5-methylcytosine (m^5^C), 5-hydroxymethylcytosine, and 7-methylguanine (m^7^G).

As for miRNAs, the existing studies have shown that miRNA methylations, mainly including m^6^A, m^5^C, and m^7^G, can regulate the expression, biological activities, and mode of action of miRNAs providing miRNAs to regulate target genes more flexibly. This paper provides insights into the current research status of methylation modifications of miRNA mentioned above and highlights potential directions for future research.

## m^6^A modification of miRNA

### Molecular mechanisms of m^6^A modification

**Figure 2:**
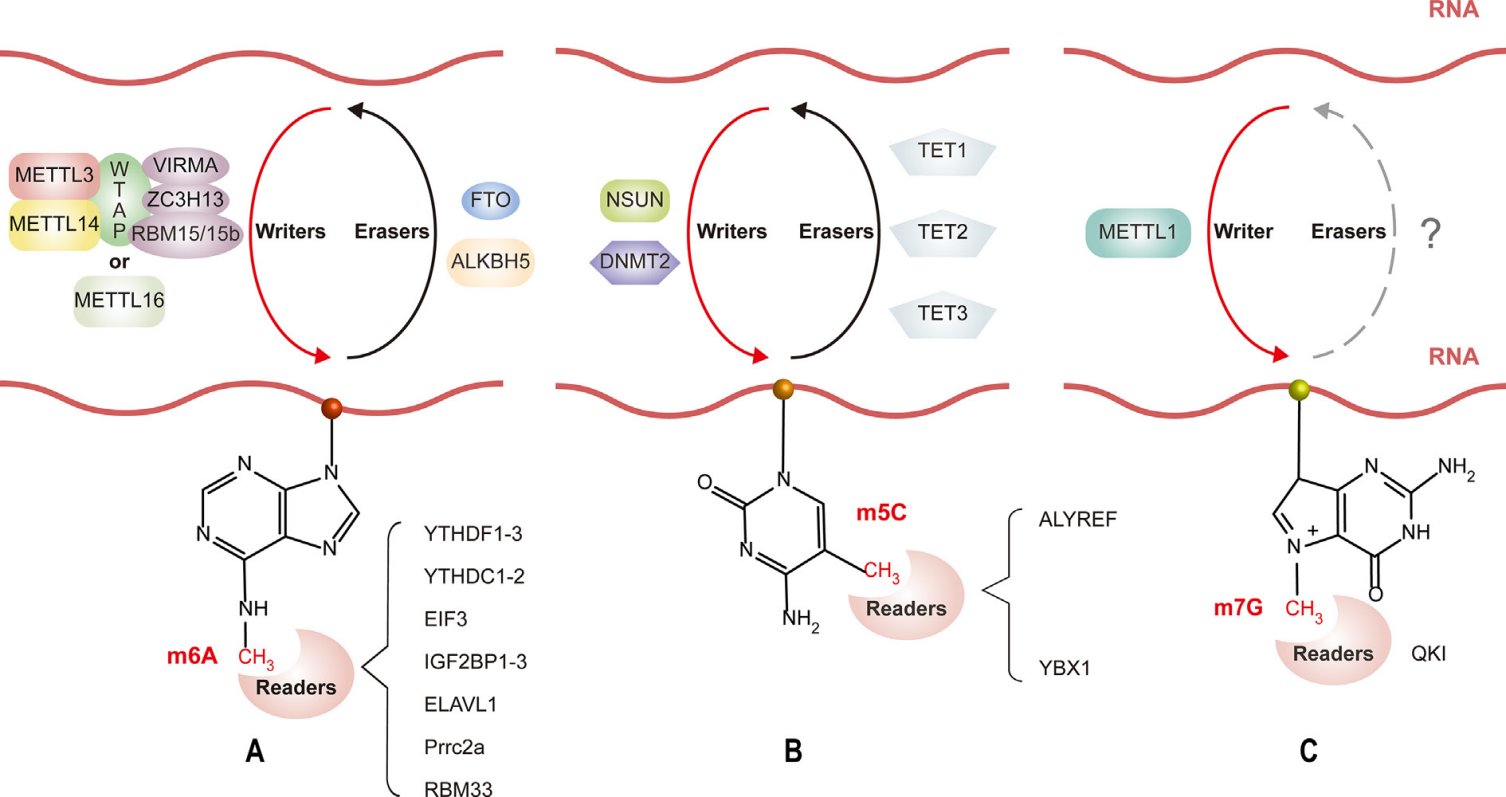
Dynamic reversible process of m^5^C, m^6^A, and m^7^G modification of RNA. Writers, erasers, and readers catalyze those modifications. **(A)** The sixth N position of adenylate can be methylated by m^6^A writers to form m^6^A. Writers include METTL3/14/16, WTAP, RBM15/15b, VIRMA, and ZC3H13. Erasers are proteins with demethylase activity and include FTO and ALKBH5. Readers are proteins that perform a biological function by recognizing m^6^A modifications, including YTHDF1–3, YTHDC1/2, eIF3, IGF2BPs, ELAVL1, Prrc2a, and RBM33. **(B)** The fifth C position of cytosine can be methylated by m^5^C writers to form m^5^C. Writers include NSUN and DNMT2. Erasers include TET1/2/3. Readers include ALYREF and YBX1. **(C)** m^7^G is a positively charged modification by METTL1, a methylated transcriptase, that co-transcriptionally modifies the methyl group at the seventh N position of the guanine. Reader includes QKI. WTAP, Wilms tumor 1 associated protein; METTL1/3/14/16, methyltransferase like 1/3/14/16; RBM15/15b, RNA binding motif protein 15/15b; RBM33, RNA binding motif protein 33; VIRMA, vir like m^6^A methyltransferase associated; ZC3H13, zinc finger CCCH-type containing protein 13; FTO, fat mass and obesity-associated protein; ALKBH5, alkB homolog 5; YTHDC1/2, YTH domain-containing 1/2; YTHDF1–3, YTH domain-containing family 1–3; ELAVL1, ELAV like RNA binding protein 1; Prrc2a, proline-rich helix-coil 2 A; NSUN, NOL1/NOP2/SUN; DNMT2, DNA methyltransferase-2; TET1/2/3, ten-eleven translocation 1/2/3; ALYREF, Aly/REF export factor; YBX1, Y-box binding protein 1; QKI, Quaking protein.

The methylation modification, often known as m^6^A, at the sixth N site of adenylate is the most common chemical modification to eukaryotic RNA (Fig. 2A). As early as 1969, Mineo Saneyoshi^45^ isolated and characterized N^6^-methyladenosine from *Escherichia coli* valine tRNA, demonstrating the existence of m^6^A modification. Then the reality of m^6^A modifications in mRNA was later identified in 1974 by Desrosiers et al.^22^ However, research on m^6^A location, regulation, and function in RNA was restricted by research techniques then.

The necessary molecular process of m^6^A modification has progressively been discovered via the advancement of m^6^A RNA sequencing and the discovery of m^6^A methyltransferases, m^6^A demethylases, and m^6^A binding proteins. Thousands of m^6^A peaks were identified precisely in the eukaryotic transcriptome, and the “DRACH” consensus motif (D = A/U/G, R = A/G, H = U/A/C) of m^6^A for Homo sapiens and Mus musculus was confirmed. Additionally, over 10,000 m^6^A peaks have been verified in more than 25% of human transcripts, and they are concentrated in long exons, close to stop codons, and 3′UTR.^39^^,^^46^

Adenosine methyltransferases (also known as “writers”) and demethylases (also known as “erasers”) regulate the reversible dynamic process of m^6^A epigenetic modification (Fig. 2A). The m^6^A modification is installed by a multicomponent methyltransferase writer complex, which consists of two subcomplexes, the m^6^A-METTL complex, which includes methyltransferase like 3 (METTL3) and METTL14, and the m^6^A-METTL associated complex, which is made up of the METTL3 adapter Wilms tumor 1 associated protein, RNA binding motif protein 15/15b, vir like m^6^A methyltransferase associated, and zinc finger CCCH-type containing protein 13.^47^ A methyl group is transferred from a donor substrate, S-adenosyl methionine, to the adenosine nucleobases in acceptor RNA substrates by the action of the m^6^A “writer” complex.^48^ Furthermore, recent research has indicated that METTL16, a homologous enzyme to METTL3, has been recognized as a m^6^A methyltransferase. Its enzymatic function is reliant on a UACAGAGAA non-polymer and a distinct RNA structure.^49^ This enzyme can introduce m^6^A modifications to both noncoding RNAs and mRNAs.^49^^,^^50^ Moreover, it has been found to play significant roles in various biological processes associated with normal development and disease.

Two distinct demethylases, including fat mass and obesity-associated protein (FTO) and alkB homolog 5 (ALKBH5), may remove m^6^A methylation, which is dynamically reversible. Jia et al^51^ showed that increased levels of m^6^A in mRNA were seen when FTO was knocked down using siRNA, but reduced levels of m^6^A *in vivo* were seen after FTO was overexpressed, demonstrating that FTO can make m^6^A revert. Thus, FTO was the first m^6^A demethylase found and a member of the ALKB family, significantly advancing the fundamental m^6^A study. ALKBH5, a different member of this family, was also defined in 2013 as a mammalian m^6^A RNA demethylase. According to research by Zheng et al,^33^ ALKBH5 catalyzed the removal of the m^6^A modification on nuclear RNA (mainly mRNA), which impacted nuclear RNA metabolism, gene expression, and RNA output. Other methylated nucleotides have minimal to no activity in the FTO and ALKBH5, which are highly selective for m^6^A.

Dynamical transcriptomic m^6^A modification is modulated by its “writers” and “erasers”. In contrast, another player in the RNA methylation game is to be required to fulfill its impact at each stage of the RNA life cycle. The m^6^A “readers” are the name given to these RNA-binding proteins (Fig. 2A). The reader proteins mainly include YTH domain-containing 1/2 (YTHDC1/2), YTH domain-containing family 1–3 (YTHDF1–3), eukaryotic translation initiation factor 3, insulin-like growth factor 2 mRNA-binding 1–3 (IGF2BP1–3), and ELAV like RNA binding protein 1. YTH family proteins have a YTH domain that acts as the module for distinguishing m^6^A from A. YTHDC1 is predominantly located in the nucleus and contributes to RNA splicing and export.^34^^,^^35^^,^^52^ The m^6^A modification site in the cytoplasm is primarily recognized and bound to by YTHDF1–3 and YTHDC2. YTHDF1 interacts with initiation factors to speed up cap-dependent translation and protein synthesis.^31^ YTHDF2, identified as the first m^6^A “reader”, regulates the degradation of the transcripts through its C-terminal region.^53^ YTHDF3 interacts with YTHDF1 to improve RNA translation and RNA degradation by enhancing the binding ability of YTHDF2 to RNA-containing m^6^A-modified substrates.^36^^,^^54^ YTHDC2 mediates mRNA degradation and improves target mRNA translation efficiency.^55^, ^56^, ^57^ In addition, proteins other than those from the YTHDF family are also known to serve as m^6^A readers. Eukaryotic translation initiation factor 3, one of the 43s translation pre-initiation complex's components, can promote translation by increasing the recruitment of the 43s complex or interacting with YTHDF1.^58^ Research on IGF2BP1-3 found that IGF2BP1-3 can enhance RNA stability.^59^ Several studies showed that ELAV like RNA binding protein 1, also known as human antigen R, improves the stability of transcripts by binding to m^6^A and mRNA.^60^, ^61^, ^62^ New m^6^A readers are also being discovered, such as proline-rich helix-coil 2 A, which controls mRNA stability by mediating m^6^A.^63^

Recent studies have debated whether heterogeneous nuclear ribonucleoprotein A2/B1 (HNRNPA2B1) is an “m^6^A reader”. According to Alarcon et al,^64^ HNRNPA2B1 mediates the alternative splicing of target RNAs and improves primary miRNA processing by directly binding to m^6^A. In contrast, Ma's group^65^ identified an HNRNPA2B1-mediated “m^6^A switch” rather than a direct binding mechanism based on protein structure analysis. In addition, two other HNRNP proteins, heterogeneous nuclear ribonucleoprotein C and heterogeneous nuclear ribonucleoprotein G, also regulate the processing of m^6^A-modified RNA transcripts.^66^^,^^67^

In recent work, Yu et al^68^ elucidated the impact of interactions between m^6^A methyltransferases and recognition proteins on the functionality of m^6^A. In their study, they have successfully identified RBM33 as a novel protein that binds to m^6^A and forms a complex with ALKBH5. This complex is responsible for the demethylation of certain transcripts by modulating the accessibility and activity of ALKBH5 substrates. The overexpression of RBM33 in human head and neck squamous cell carcinoma (HNSCC) cells has been observed to facilitate the development of HNSCC tumors by enhancing the stability of DDIT4 in a manner that is dependent on m^6^A modification. Consequently, this process leads to the induction of autophagy. This observation sheds light on the crucial oncogenic function of the RBM33/ALKBH5 signaling pathway in HNSCC, as demonstrated by various mouse tumor models, including a xenograft model utilizing patient-derived tumors. This discovery broadens the scope of the investigation into the substrate selectivity of m^6^A modification, elucidates a previously unknown mechanism through which ALKBH5 facilitates mRNA m^6^A demethylation, and identifies prospective therapeutic targets for treating HNSCC. Consequently, these findings hold promise for future clinical applications.^68^

In conclusion, the destiny of the changed transcripts and the phenotypic effects on the cell depends on the coordinated action of m^6^A “writers”, “erasers”, and “readers".

### Biological functions of miRNA m^6^A modifications

**Figure 3:**
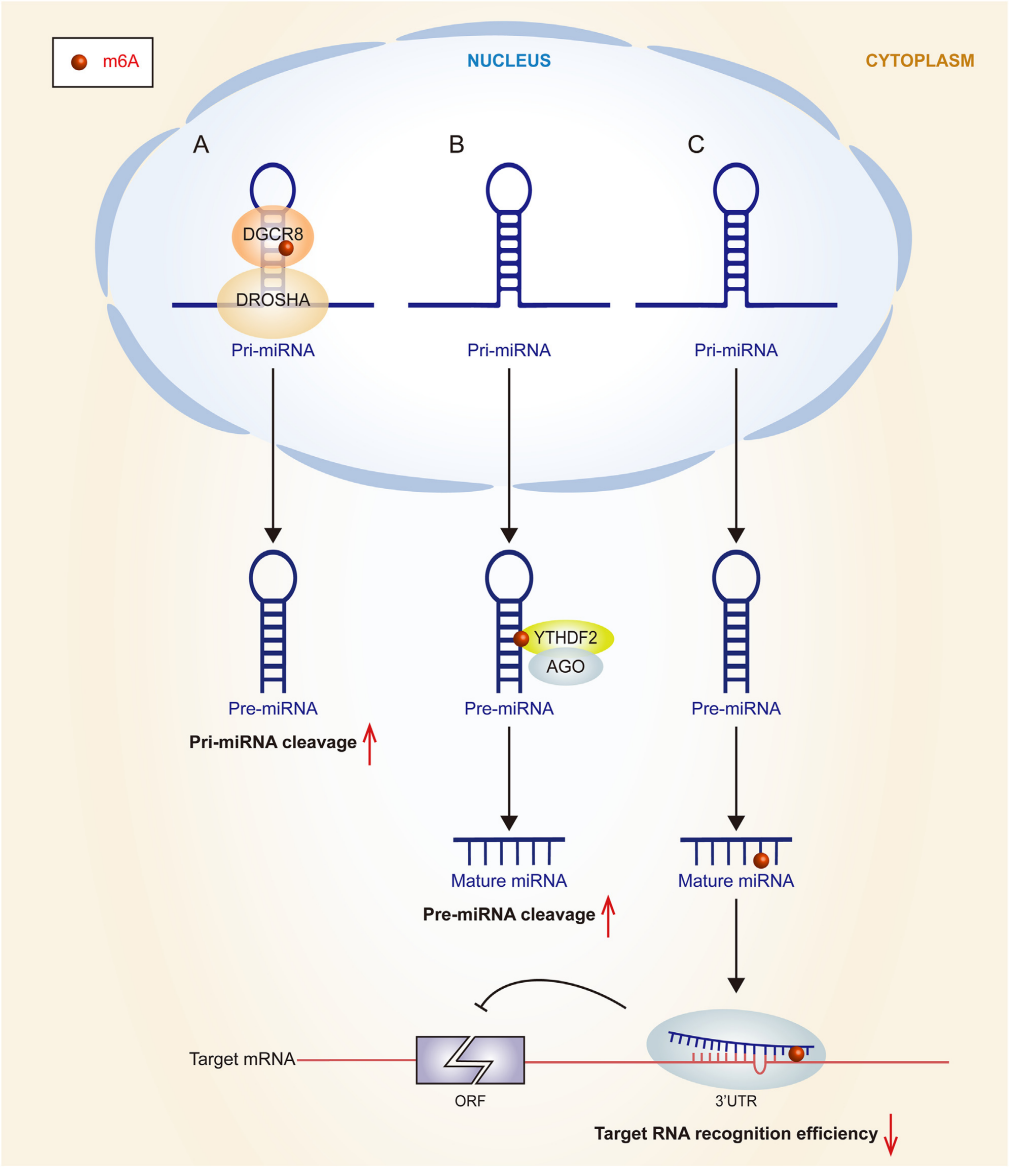
Biological functions of miRNA m^6^A modifications. **(A)** In the nucleus, m^6^A modification contributes to recruiting DGCR8 to target pri-miRNA, thus facilitating the cleavage of pri-miRNA to pre-miRNA by DGCR8 and the intranuclear enzyme Drosha. **(B)** In the cytoplasm, m^6^A modifications on pre-miRNAs are recognized by YTHDF2, which then recruits the AGO2 protein to help pre-miRNAs to shear and promote their maturation. **(C)** The m^6^A modification on miRNA resulted in significant structural changes of miRISC complexes, thereby repressing the target mRNA recognition efficiency. DGCR8, DiGeorge syndrome chromosomal region 8; YTHDF2, YTH domain-containing family 2; AGO2, Argonaute 2; miRISC, miRNA-induced silencing complex.

Numerous studies in recent years have amply demonstrated that m^6^A modification can influence miRNA function in several ways, with the regulation of miRNA biosynthesis attracting the most attention.^69^ m^6^A modifications are associated with the accurate recognition and binding of pri-miRNA by DGCR8 from numerous transcripts with secondary structure during miRNA biosynthesis molecular mechanism (Fig. 3A).

In 2014, Yuan et al^70^ demonstrated that tRNA methyltransferase NOP2/Sun RNA methyltransferase 2 (NSun2) methylates miR-125b at different stages *in vitro* and *in vivo*. This, in turn, prevents the conversion of pri-miR-125b-2 into pre-miR-125b-2 and the cleavage of pre-miR-125b-2 into miR-125 and lowers the recruitment of RISC by miR-125b, which restricts the ability of miR-125b to silence its target mRNA. However, there is now a debate about the role of NSun2 as a methylation transferase for m^6^A. Contrary to the assertion made in this work that NSun2 functions as a “writer” of m^6^A, later research has predominantly characterized NSun2 as a “writer” of m^5^C.^32^ However, there is currently insufficient conclusive evidence to definitively classify NSun2 as a methylation transferase responsible for m^6^A modification. Despite the existence of certain controversies, this particular work stands as the earliest investigation within the literature that explores the involvement of m^6^A methylation in the processing and maturation of miRNA.

As mentioned above, the way that DGCR8 preferentially identifies and binds to the secondary structures of the pri-miRNAs present in transcripts still needs to be fully elucidated. Tavazoie's group^42^ conducted an in-depth study and exploration of this issue in 2015. They demonstrated that METTL3 methylates primary intergenic and intragenic miRNAs and makes it easier for DGCR8 to detect and attach to its substrate, thereby facilitating the onset of miRNA synthesis. In the same year, the involvement of HNRNPA2B1 in pri-miRNA processing was demonstrated. Similar to the m^6^A “writer”, METTL3 interacts with the DGCR8 to directly bind to pri-miRNAs and modify their alternative splicing.^64^ Additionally, a group of miRNAs whose processing depends on both METTL3 and HNRNPA2B1 suggests that HNRNPA2B1 partially mediates the impact of m^6^A/METTL3 on miRNA processing.^64^

Furthermore, types of research have demonstrated that m^6^A modification of pri-miRNAs is essential for understanding clinical disease pathogenesis and treatment. It has been reported that by up-regulating METTL3 expression, cigarette smoke condensate promotes the m^6^A level of pri-miR-25 and enhances the production of mature miR-25-3p in pancreatic duct epithelial cells. Consequently, more mature miR-25-3p significantly reinforces the simulation of carcinogenic AKT-p70S6K signaling, which encourages the development of pancreatic cancer and is linked to an unfavorable prognosis in pancreatic cancer patients.^71^ In addition to m^6^A “writers”, “reader” proteins may also be involved in the pathogenesis of diseases. It has been discovered that RALY is a crucial regulator of the Drosha complex. Through the m^6^A switch, RALY increases the production of miR-877, miR-676, and miR-483 at the post-transcriptional stage. These miRNAs specifically inhibit the expression of the metabolism-related genes ATP5I, ATP5G1, ATP5G3, and CYC1, altering mitochondrial metabolism and promoting the formation and progression of colorectal tumors in both *in vivo* and organoid models.^72^

Besides promoting the pri-miRNA processing, m^6^A modifications in pre-miRNAs can also affect miRNA maturation (Fig. 3B). In the latest study, Zhang et al^73^ found that YTHDF2 is aberrantly expressed in acute myeloid leukemia patients with oncogenic effects. The YTHDF2 protein mechanistically recognizes the m^6^A modification present in pre-miR-126 and then binds to AGO2 to facilitate the maturation process of pre-miR-126. This process results in the establishment of a positive correlation between YTHDF2 and miR-126, as well as a negative correlation between YTHDF2 and downstream target genes of miR-126 in patients with acute myeloid leukemia. This finding suggests a role for m^6^A in pre-miRNA processing that contributes to tumorigenesis and reflects the therapeutic potential of targeting YTHDF2/miR-126 for acute myeloid leukemia treatment.^73^ The aforementioned discovery implies that m^6^A plays a part in the processing of pre-miRNA, which leads to the development of tumors. Additionally, it highlights the potential therapeutic value of targeting YTHDF2/miR-126 for acute myeloid leukemia. While the mechanism behind the involvement of m^6^A modifications in pre-miRNA cleavage has been investigated, the understanding of the biological implications of this mechanism in disease is comparatively limited in comparison to the well-studied role of m^6^A modifications in pri-miRNA cleavage. Thus, additional exploration is necessary to elucidate the precise function of this mechanism in disease contexts.

Additionally, m^6^A modification can occur to mature miRNAs (Fig. 3C) and has the ability to reduce the miRNA's ability to inhibit the translation of target mRNAs. Konon et al^74^ identified m^6^A modifications in mature miR-17-5p and let-7a-5p by a non-targeted mass spectrometry sequencing method, and their sites were not near the RNA binding site. Later, by a molecular mechanics approach, they predicted that m^6^A modification led to significant structural changes in the miRNA-induced silencing complex, including the RNA recognition site, which affects the target mRNA recognition efficiency. Thus, m^6^A modification can promote mRNA expression levels by inhibiting miRNA recognition of miRNAs with mRNAs and inhibiting the ability of miRNAs to attenuate the translation of target mRNAs.^74^

Moreover, the presence of m^6^A modifications on the target mRNA can potentially influence the binding between the mRNA and miRNA, thereby impacting the regulatory role of the miRNA. One potential consequence of m^6^A modifications occurring within miRNA binding sites is the potential direct impact on the stability of miRNA-mRNA double-stranded structures. This, in turn, could lead to a decrease in the post-transcriptional repression exerted by miRNAs. In cancer cell lines, the m^6^A modification has been observed to facilitate the recruitment of IGF2BP1 to the 3′UTR region of serum response factor mRNA. This recruitment subsequently leads to a decrease in the binding of AGO to serum response factor, hence interfering with the miRNA-mediated binding process. Therefore, the m^6^A on the 3′UTR of serum response factor mRNA mitigated the inhibitory post-transcriptional control exerted by miRNAs. On the other hand, m^6^A modifications occurring inside the binding sites of miRNAs have the potential to modify miRNA targeting through the modulation of mRNA secondary structure or the recruitment of other proteins. Through sequence alignment analysis, Qian et al^75^ found that among 139 cardiac miRNAs, only the seed sequence of miR-133a was inversely complemented to the m^6^A consensus motif “GGACH”. Further studies revealed that IGF2BP2 could bind to the m^6^A-modified site on the 3′UTR of the miR-133a target mRNAs and interact with AGO2. Thus, it promotes the accumulation of miR-133a-AGO2-RISC complex on its target and enhances the decrease of target mRNA stability and translation.^75^ In general, the aforementioned instances underscore the ambiguity surrounding the impact of m^6^A modifications in mRNA 3′UTRs on miRNA targeting.

In general, it can be stated that m^6^A modification is a dynamic and reversible epigenetic modification of miRNAs. Moreover, it plays a critical role in various aspects of miRNA biosynthesis and function, such as miRNA processing, mRNA-miRNA interactions, and m^6^A target selection (Table 1). Furthermore, the m^6^A modifications of miRNAs in the context of disease possess the capacity to impact the progression of said disease via the several pathways elucidated before. In the majority of these mechanisms, the regulatory process of m^6^A modification of miRNAs initiates with alterations in the expression or functionality of the enzymes accountable for the addition or elimination of methyl groups. These modifications subsequently result in abnormal levels of methylation, which consequently induce the dysregulation of miRNA expression levels. Subsequent alterations result in abnormal methylation levels, thus leading to the dysregulation of miRNA expression levels. For example, in bladder, colorectal, lung, ovarian, and gallbladder malignancies, the up-regulation of METTL3 has been observed to facilitate the maturation of many miRNAs, including miR-221/222,^76^ miR-1246,^77^ miR-126-5p,^78^ and miR-92.^79^ Furthermore, the aggregation of these miRNAs, which are dependent on m^6^A modification, facilitates the advancement of tumors. Consequently, the possibility of targeting the m^6^A “writers” or “erasers”, disrupts this process and enables the treatment of the disease.

**Table 1:** Mechanisms of methylation modification of miRNAs in disease processes.

Modification Type	Regulatory Mechanisms	Modified miRNA(s)	Types of diseases	Effects on disease progression	Reference
m^6^A	Mediating processing of pri-miRNAs	miR-25-3p	Pancreatic cancer	Promotion of miR-25-3p maturation leads to a decrease in PHLPP2, which results in AKT activation.	71
		miR-221/222	Bladder cancer	Promotion of miR-221/222 maturation leads to a decrease in PTEN, which leads to tumor proliferation	76
		miR-1246	Colorectal cancer	Promotion of miR-1246 maturation leads to a decrease in SPRED2, which activates the RAF/MEK/ERK pathway	77
		miR-126-5p	Ovarian cancer	Promotion of miR-126-5p maturation leads to a decrease in PTEN, which activates the PI3K/Akt/mTOR pathway	78
		miR-92	Gallbladder cancer	Up-regulation of mature miR-92 results in the reduction of PTEN, thus activating PI3K/AKT signaling	79
	Mediating processing of pre-miRNAs	miR-126	Acute myeloid leukemia	YTHDF2 protein recognizes the m6A modification present in pre-miR-126, which then binds to AGO2 and facilitates the maturation process of pre-miR-126, thereby promoting tumorigenesis.	73
	Influence on miRNAs targeting	miR-17-5p	Colorectal Cancer/Pancreatic cancer	n.d.	74

		let-7a-5p	Colorectal Cancer/Pancreatic cancer	n.d.	74
m^5^C	Influence on miRNA targeting	miR-200c	Gastrointestinal cancers	n.d.	74
	Mediating processing of pri-miRNAs	miR-181a-5p	Glioma	The m^5^C modification of mature miR-181a-5p results in the loss of its ability to target mRNA for the pro-apoptotic protein BIM	98
m^7^G	Mediating processing of pri-miRNAs	let-7e-5p	Colon cancer	Mediates let-7 maturation to activate its target HMGA2, thereby enhancing colon cancer cell viability and mobility	108

Considering the significant involvement of m^6^A regulatory proteins in diverse pathological conditions, the exploration of small molecule inhibitors or agonists that specifically target dysregulated m^6^A regulatory proteins holds great potential as viable therapeutic options, particularly in the realm of cancer treatment. Several small molecule drugs, namely Rhein, MO-I-500, and MA/MA2, were previously developed to specifically target FTO.^80^ In recent years, a newly discovered FTO inhibitor called FB23-2 has emerged.^81^ This chemical has demonstrated the ability to impede the proliferation of acute myeloid leukemia cells while also promoting their differentiation.^81^ Similarly, ALK-04 was formulated to target ALKBH5,^82^ STM2457 was designed to counteract METTL3,^83^ and BTYNB and CWI1-2 were produced to inhibit IGF2BP1-2.^84^^,^^85^ Despite the significant advancements in research in recent years, the development of medicines focusing on m^6^A modification in cancer is still in its early stages. Potential avenues for future research encompass, however, are not confined to the elucidation of additional m^6^A readers capable of discerning m^6^A modifications inside pri-miRNAs, pre-miRNAs, mature miRNAs, or their respective target sequences, hence facilitating diverse biological processes. The inclusion of clinical validation about small molecule medications created by the targeting of m^6^A-modified proteins is also encompassed. The simultaneous initiation of basic research and clinical trials will effectively facilitate the advancement of precision medicine through the utilization of RNA treatment.

## m^5^C modification of miRNA

### Molecular mechanisms of m^5^C modification regulation

m^5^C, a modification of the fifth C position of cytosine by methylation (Fig. 2B), was the first modification found on a nucleotide and is a frequent chemical modification in DNA. It was reported in tRNA, rRNA, and mRNA as early as the 1970s.^22^^,^^86^ Unfortunately, little advancement has occurred in the distribution and biological effects of m^5^C modification in RNA because of the need for precise and practical techniques for RNA methylation detection. With the development of bisulfite sequencing, m^5^C-RIP-seq,^87^ Aza-IP-seq,^88^ and miCLIP-seq,^89^ the transcriptome-wide mapping of m^5^C can be precisely identified at single-nucleotide resolution.

Like m^6^A, m^5^C modification is also a reversible dynamic regulatory process and has its own “writers”, “erasers”, and “readers” (Fig. 2B). m^5^C methylated transferase also uses S-adenosyl methionine as a methyl donor to add methyl to cytosine to form 5-methylcytosine.^90^ Currently, as many as 10 different types of RNA m^5^C methyltransferases have been ascertained, including NOL1/NOP2/SUN (NSUN) domain family members^90^ and DNA methyltransferase-2.^91^ The m^5^C demethylated transferases, which are members of the ten-eleven translocation family, are dioxygenases that require Fe(II) and 2-oxoglutarate to catalyze the conversion of m^5^C to 5-hydroxymethylcytosine and then to 5-formylcytosine.^92^ Members of this family include ten-eleven translocation 1/2/3, which are active against 5-methylcytidine and its oxidized analogs in RNA, and the ten-eleven translocation enzyme family catalyzes the synthesis of dsDNA, ssDNA, ssRNA, and DNA-RNA hybrid strands.^92^, ^93^, ^94^, ^95^ RNA m^5^C methylated recognition proteins, such as Aly/REF export factor and Y-box binding protein 1, have been validated to be m^5^C readers and play a crucial part in mRNA transit and stability.^32^

### The biological function of miRNA m^5^C modification

It has been verified that the m^5^C modifications are prevalent in miRNA, tRNA, rRNA, tRNA-derived small RNAs (tRNA-derived small RNAs), and rRNA-derived small RNAs (rsRNA-28S).^22^^,^^44^^,^^88^^,^^96^ The first high-throughput next generation sequencing-based method (BS-miRNA-seq) and an analysis pipeline (MAmBA) to achieve high-resolution mapping of m^5^C modifications were described in 2021.^97^ Furthermore, using those technologies, they discovered m^5^C and 5-hydroxymethylcytidine sites in mature miRNAs.^97^ The advancement of those methods has made it possible to identify m^5^C on miRNAs in several cell types and tissues, which is a crucial initial step in comprehending the modifying roles of miRNAs (Table 1). So far, few studies have reported the functions of miRNA m^5^C modification. Konon et al^74^ produced miR-200c oligonucleotides with m^5^C modifications at all cytosines, which was used to determine the effects on its function. The findings demonstrate that, in miR-200c-3p, methyl groups of the cytosine at position 9 that are near RNA recognition bases disrupt hydrogen bonding with Ser220 of AGO, most probably through steric hindrance, which is supposed to lead to a positional change of the guanine at position 8 that is also caused by interaction with Arg761 of AGO.^74^ In 2020, according to research by Cheray's team,^98^ a sizable portion of miRNAs was m^5^C-containing. miRNAs' m^5^C methylation, catalyzed by DNA methyltransferase 3 alpha and AGO4 complex, reduces the production of miRNA/mRNA duplexes, resulting in the loss of their ability to suppress the expression of their target mRNAs. Moreover, m^5^C of miR-181a-5p eliminates its tumor suppressor activity, giving patients with glioblastoma multiforme a lousy prognosis.^98^

## m^7^G modification of miRNA

### Molecular mechanisms and biological functions of the regulation of m^7^G methylation modifications

m^7^G modification, a common RNA modification in post-transcriptional regulation, is widely found at the 5′ caps and inner locations of mRNA,^99^ rRNA,^100^ and tRNA^101^ of all eukaryotic species. m^7^G is a positively charged modification by METTL1 complex, a methylated transcriptase, that co-transcriptionally modifies the methyl group at the seventh N position of the 5′ cap guanine (G) (Fig. 2C).^102^ As early as 1975, scientists detected m^7^G modification in mRNA and later found that it had various biological functions.^103^ It is essential for RNA stability,^104^ nuclear export,^105^ and protein translation.^103^ While the discovery of the biological activity of m^7^G occurred in the early stages, it is only in recent times that researchers have successfully identified readers of m^7^G. In a work conducted by Zhao et al,^106^ the authors identified Quaking proteins (QKIs) that have the ability to specifically identify m^7^G modifications on mRNA. QKI belongs to the STAR (signal transduction and activation of RNA metabolism) family of RBPs with K homology domain.^107^ In situations of heightened stress, the QKI protein variant QKI7 engages in interactions with m^7^G-modified mRNAs, facilitating their sequestration into stress granules. Additionally, QKI7 has the capability to directly connect with the core protein G3BP1 of the stress granules, thereby exerting an influence on the stability and/or efficiency of mRNA translation. Furthermore, it has been observed that QKI7 exerts an inhibitory effect on the Hippo signaling pathway utilizing a m^7^G-dependent mechanism. This phenomenon renders cancer cells more susceptible to the effects of chemotherapeutic drugs.^106^

However, due to a lack of sensitive detection technology, the identification of m^7^G inside low-abundance RNA transcripts has been hindered. It was in 2019 that Pandolfini et al^108^ established a chemical reactivity assay, borohydride reduction sequencing, to detect internal m^7^G in miRNAs. Using RNA immunoprecipitation and borohydride reduction sequencing, they identified METTL1-dependent m^7^G within a subset of miRNAs. Furthermore, they applied a more refined mass spectrometry approach to identify m^7^G within the let-7e-5p miRNA at guanosine 11. They found that m^7^G modification facilitated the processing of miRNA by disrupting the inhibitory secondary structure within pri-miRNA and promoting its formation into a G-quadruplex for Drosha cleaving.^108^

However, there is some controversy about this finding, mainly focusing on the presence of m^7^G modifications within let-7e. Vinther's team^109^^,^^110^ found that an RNA segment that matches the let-7e fragment in the sequence is generated by cleavage of human LSU rRNA by nuclear ribonuclease A. Surprisingly, this segment exhibits a U60 snoRNA-guided 2′-O-Me modification exactly where Pandolfini claimed there to be an m^7^G modification.^108^ Furthermore, they demonstrated that the methylation of let-7e is not supported by their data from human HeLa cells.^111^ Therefore, they concluded that let-7e does not have m^7^G modification and Pandolfini's liquid chromatography-mass spectrometry and mass spectrometry results were not credible.^112^ In response to this skepticism, Pandolfini provided a refutation, contending that the issue was brought on by Vinthe's technique's lack of sensitivity and the lower degrees of modification in HeLa cells.^113^ Further research needs to clarify that METTL1-mediated miRNA m^7^G modification modulates its structure and biogenesis.

Similarly, m^7^G modifications in miRNAs must also be a dynamic and reversible biological process (Fig. 2C) and have implications for disease development (Table 1). Hence the regulators of m^7^G miRNA methylation need to be clarified to fill in the gaps in m^7^G biogenesis in miRNAs in future studies.

## Conclusions

In summary, methylation modification plays an important regulatory role for miRNA biogenesis, structural stability, and biological functions, which adds a new dimension to the understanding of miRNA biology. Thus, the significance of studying the methylation modification of miRNAs in depth is extensive and far-reaching. For one, it helps to reveal miRNAs' molecular mechanism and mode of action. Second, it provides a new entry point for people to deeply study the mechanisms of different biological processes and disease development. Third, the methylation modifications of miRNA have excellent potential for application as biomarkers for early clinical diagnosis, recurrence, treatment effect, and illness prognosis, and even for therapeutic targets and the development of specific target drugs.

Despite the encouraging results of the above studies, there are still many questions about the study of miRNA methylation modifications. The first is about the detection method of methylation modification. The current mainstream detection method, methylated RNA immunoprecipitation with next-generation sequencing, cannot achieve single nucleotide localization analysis, and it is challenging to locate the methylation sites precisely. This technique has the limitation of modification species detection. Specific technology is required for m^5^C and m^7^G modifications, such as BS-miRNA-seq and borohydride reduction sequencing mentioned above. Although there is some controversy as to whether the sensitivity of the borohydride reduction sequencing technique can detect m^7^G modifications on miRNAs, existing results demonstrate the need for more accurate and sensitive technology. If specific technical barriers can be overcome, and the methods can be widespread, the study of m^5^C and m^7^G-related miRNA modifications will be more in-depth, and some controversies can be resolved. Secondly, there is still more to be done regarding methylation data analysis techniques and applications. For instance, the growing body of information on miRNA methylation modifications necessitates the creation of a database that can facilitate the sharing and extensive use of pertinent data and may even help predict the locations and purposes of miRNA methylation modifications and analyze and summarize the connection between miRNA methylation modifications and illness incidence. Finally, the research on the methylation modification of miRNAs currently focuses on their expression changes under different physiological and pathological conditions. However, if methylation modification of miRNAs is to be applied as diagnostic markers or clinical therapeutic targets, further investigation of the details of the relevant mechanism is required. In addition, molecules related to the methylation modification of miRNAs still need to be further studied and explored. All of them may affect the regulation of miRNA expression on target genes, which will be an essential direction for developing miRNA-related clinical technology tools in the future.

## Author contributions

Ning Su and Xiaohang Yu drafted the manuscript. Ming Duan and Ning Shi revised the manuscript for important intellectual content. All authors read and approved the final manuscript.

## Funding

This work was supported by the 10.13039/100007847Natural Science Foundation of Jilin Province, China (No. 20230101147JC); the 10.13039/501100001809National Natural Science Foundation of China (No. 31970154); and the 10.13039/501100002858China Postdoctoral Science Foundation (No. 2022TQ0119).

## Conflict of interests

The authors declared that there was no conflict of interests.
